# Metagenomic and genomic sequences from a nitrate-reducing benzene-degrading enrichment culture

**DOI:** 10.1128/mra.00294-24

**Published:** 2024-09-09

**Authors:** Johnny Z. Xiao, Camilla L. Nesbø, Olivia Molenda, Courtney R. A. Toth, Elizabeth A. Edwards

**Affiliations:** 1 Department of Chemical Engineering and Applied Chemistry, University of Toronto, Toronto, Ontario, Canada; 2 Department of Biological Sciences, University of Alberta, Edmonton, Alberta, Canada; California State University San Marcos, San Marcos, California, USA

**Keywords:** metagenomes, anaerobic, benzene, nitrate reduction, assembled genomes

## Abstract

Metagenome-assembled genomes (MAGs) were recovered from metagenomic assemblies from a nitrate-reducing benzene-degrading enrichment culture. Ten MAGs of high quality or functional interest to benzene degradation are reported, seven of which are single contig genomes.

## ANNOUNCEMENT

Anaerobic benzene biodegradation has been shown in several mixed enrichment cultures, yet the mechanism(s) of biochemical benzene activation remains elusive (1–3). Our laboratory maintains a nitrate-reducing benzene-degrading culture (NRBC) established in 1995 from a decommissioned gas station in Toronto, Canada (latitude: 43.722647, longitude: −79.463022) (1, 2). The NRBC culture, previously called Cartwright-NO_3_ (3), is grown in a defined anaerobic mineral medium amended with benzene (300–400 µM) and nitrate (~2 mM) (4). Metatranscriptomic analysis of this culture performed over a decade ago suggested that a member of the family Peptococcaceae (now resolved to be a *Thermincola*) was responsible for initiating benzene metabolism (5), in agreement with more recent results (3). The microbes in NRBC are poorly understood with no isolated representatives; therefore, metagenomic sequencing was conducted to shed light on their functions.

**Fig 1:**
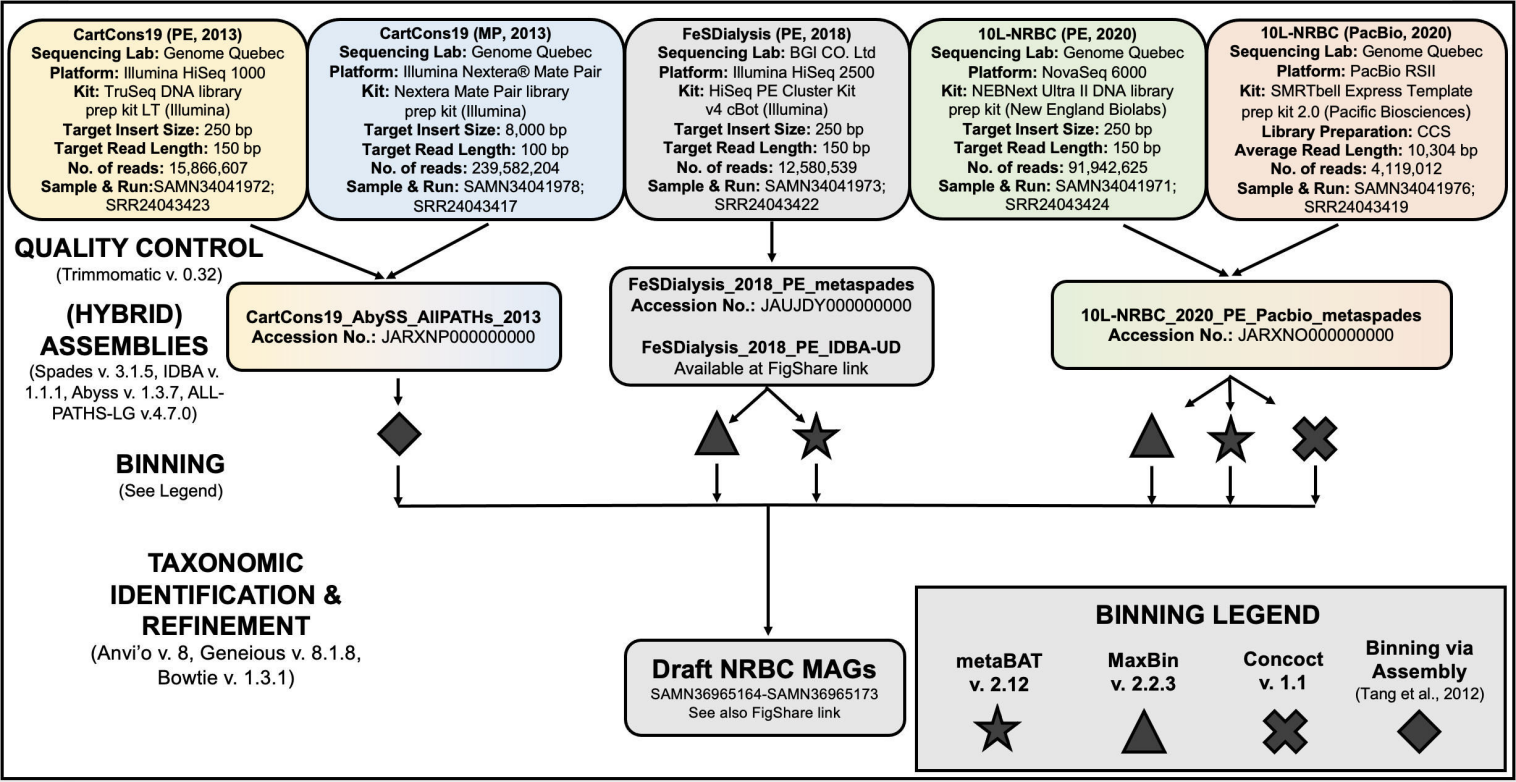
Metagenomic workflow used for (hybrid) assembly, binning, and curation of draft NRBC metagenome-assembled genomes (MAGs). Default parameters were used for all software unless otherwise specified. Sequencing labs were the Centre d’expertise et de service Génome Québec (Montreal, Canada) and BGI Genomics (China).

DNA was extracted from samples (15 mL) of three NRBC subculture bottles in 2013 (CartCons19), 2018 (FeS-Dialysis), and 2020 (10L-NRBC) using the DNeasy PowerSoil Kit (Qiagen). At the time of sampling, the volume of each culture ranged from 0.5 to 10 L, and benzene degradation rates varied from 0.5 to 4.0 mg/L/day. Each DNA sample was sequenced once or twice using Illumina or PacBio technology with library preparation approaches and kits listed in Fig. 1. For Illumina sequencing, kits were all used according to the manufacturer’s instructions. Library preparation for PacBio sequencing used the SMRTbell Express Template Prep Kit 2.0 (Pacific Biosciences) without size selection. Adaptor sequences and low-quality bases were removed using Trimmomatic v. 0.32 (6). Illumina paired-end (PE) and mate-pair (MP) 2013 sequencing data from CartCons19 were processed using AbySS v. 1.3.7 (7) to create unitigs, which were merged with scaffolds generated in ALL-PATHS-LG v. 4.7.0 (8) using gap-filling Perl scripts published in Text S1 of Tang et al. (9). PE reads from the FeS-Dialysis metagenome were assembled using SPAdes v. 3.10.1 (10) or IDBA v. 2018 (11), then binned using metaBAT v. 2.12 (12) and MaxBin v. 2.2.7 (13) with default settings. PE and PacBio reads from the 10L-NRBC metagenome were co-assembled using SPAdes v. 3.15.3 and binned using MaxBin v. 2.2.7 (13), metaBAT v. 2.12 (12), and Concoct (14) with default settings (Fig. 1).

A total of 868 bins were compared and dereplicated using dRep v. 2.0.0 with default settings (15) yielding 79 unique metagenome-assembled genomes (MAGs). Illumina reads were mapped to the dereplicated MAGs using the Anvi’o v.8 metagenome-snakemake-workflow to obtain completeness, abundance, and coverage estimates (16). Taxonomy was assigned using the Silva database (v138.1 SSU) if a 16S rRNA gene was identified (17) and/or using release 214 of the Genome Taxonomy Database (GTDB) and GTDB tool kit (v. 2.3.2, classify_wf command) (18, 19). Ten highly contiguous MAGs that were either of high quality (low contamination, >95% completeness, including 23S, 16S, and 5S rRNAs and >23 tRNA genes) or of medium quality but of interest due to the association with anaerobic benzene catabolism (3) were then manually curated using Bowtie v. 1.3.1 (20) to resolve ambiguities and then further refined using the *de novo* assembly tool in Geneious v. 8.1.8 (21). The metagenomes were annotated using MetaErg v. 1.2.3 (22), and the MAGs were submitted to NCBI and annotated using the Prokaryotic Genome Annotation Pipeline (23). The details for each of the 10 refined MAGs including quality and taxonomy are summarized in Table 1. A large fraction of metagenomic reads (40%–70%) were mapped to these 10 MAGs (see FigShare). The MAGs include predicted denitrifiers (*Aromatoleum*, *Denitratisoma*), taxa of unknown function, and the putative benzene degrader, *Thermincola*. Additional work to refine the *Thermincola* MAG is described separately (24).

**TABLE 1:** Assembly information of 3 metagenomes and 10 MAGs from NRBC enrichment cultures, including accession numbers^*e*^

Metagenomic assembly/MAG name	Length (Mb)	No. of contigs or scaffolds	GC content (%)	Average coverage or read depth	MAG completeness (%)^*a*^	MAG contamination (%)^*a*^	NCBI accession no.
CartCons19 PE and MP AbySS-ALLPATHs 2013 (**Assembly #1**)	211	25,984	59.5	47	NA	NA	JARXNP000000000.1
FeSDialysis 2018 PE metaspades (**Assembly #2**)	89	25,511	59.0	219	NA	NA	JAUJDY000000000.1
10LReactor 2020 PE and Pacbio HybridSpades (**Assembly #3**)	347	28,928	63.5	342	NA	NA	JARXNO000000000.1
***Aromatoleum* sp.**^*b*^ (from Assembly #2)	4.79	47	66.5	31.3	97.3	1.52	JAVSMM000000000.1
***Burkholderiaceae* sp.**^*b*^ (from Assembly #1)	4.01	1	68.0	40.8	95.8	5.63	JAVSMN000000000.1
***Denitratisoma* sp.**^*c*^ (from Assembly #1)	3.41	1	66.1	15.86	98.6	1.41	JAVSMO000000000.1
***Ignavibacterium* sp.**^*c*^ (from Assembly #3)	3.66	1	33.5	91.5	100	1.41	JAVSMP000000000.1
***Kapabacteria* sp.** (from Assembly #1)	4.53	1	34.5	10.46	95.7	0	JAVSMQ000000000.1
***Pseudomonadaceae* sp.** (from Assembly #1)	4.12	1	64.5	2.95	98.6	1.41	JAVSMR000000000.1
***Pseudorhodoplanes* sp.** (from Assembly #1)	4.97	1	61.7	5.52	98.6	2.82	JAVSMS000000000.1
***Thiobacillus* sp.**^*b*^ (from Assembly #3)	3.37	10	64.0	12.03	92.4	9.34	JAVSMT000000000.1
***Truepera* sp.**^*c*^ (from Assembly #1)	2.92	1	67.8	9.54	94.7	0	JAVSMU000000000.1
***Thermincola* sp.**^*b*^^*,*^^*c*^ (from FeSDialysis 2018 PE IDBA-UD Assembly^*d*^)	3.83	157	44.7	6.08	96.9	2.68	JAVSMV000000000.1

^*a*^ Completeness and contamination statistics are from NCBI (if available) or from Anvi’o. NA, not applicable.

^*b*^ Indicates four MAGs of medium quality. All other MAGS are of high quality.

^*c*^ Indicates four MAGs that were annotated with the Silva database from 16S rRNA sequences. All others were annotated using GTDB.

^*d*^ This assembly (FeSDialysis 2018 PE IDBA-UD) is available on Figshare, not NCBI, as it is very similar to Assembly #2.

^*e*^ Bold signifies the connection between individual metagenome-assembled genomes and their respective metagenome assemblies.

## Data Availability

Illumina and PacBio reads are available at NCBI under project PRJNA951427, specifically under accession numbers SRR24043423 (paired-end, 2013), SRR24043417 (mate-pair, 2013), SRR24043422 (paired-end, 2018), SRR24043424 (paired-end, 2020), and SRR24043419 (PacBio, 2020). The accession numbers for the best metagenomic assemblies and MAGs derived from these sequence reads are provided in Table 1. An additional assembly and assembly details, gap-filling perl script, FASTA files and relevant statistics for the set of 79 dereplicated MAGs, and their GTDB phylogenomic classifications are available on Figshare (https://doi.org/10.6084/m9.figshare.22637596.v4). The same *Aromatoleum* sp. MAG is also available in US DOE Joint Genome Institute’s Integrated Microbial Genomes (IMG) system under the taxon ID 2886190707.
